# Anti-Hyperglycemic Effects of Refined Fractions from *Cyclocarya paliurus* Leaves on Streptozotocin-Induced Diabetic Mice

**DOI:** 10.3390/molecules26226886

**Published:** 2021-11-15

**Authors:** Zheling Feng, Zhujun Fang, Cheng Chen, Chi Teng Vong, Jiali Chen, Ruohan Lou, Maggie Pui Man Hoi, Lishe Gan, Ligen Lin

**Affiliations:** 1 State Key Laboratory of Quality Research in Chinese Medicine, Institute of Chinese Medical Sciences, University of Macau, Avenida da Universidade, Taipa, Macao 999078, China; yb77508@um.edu.mo (Z.F.); mb85812@um.edu.mo (C.C.); gigict.vong@gmail.com (C.T.V.); yc07516@um.edu.mo (J.C.); mc05836@um.edu.mo (R.L.); MagHoi@um.edu.mo (M.P.M.H.); 2 Department of Clinical Pharmacy, Zhejiang Provincial Key Laboratory for Drug Evaluation and Clinical Research, The First Affiliated Hospital, College of Medicine, Zhejiang University, Hangzhou 310000, China; 1320127@zju.edu.cn; 3 School of Biotechnology and Health Sciences, Wuyi University, Jiangmen 529020, China

**Keywords:** *Cyclocarya paliurus*, flavonoids, polysaccharides, triterpenoids, anti-hyperglycemia

## Abstract

To identify the chemical components responsible for the anti-hyperglycemic effect of *Cyclocarya paliurus* (Batal.) Iljinsk (Juglandaceae) leaves, an ethanol extract (CPE) and a water extract (CPW) of *C. paliurus* leaves, as well as their total flavonoids (CPF), triterpenoids (CPT) and crude polysaccharides (CPP), were prepared and assessed on streptozotocin (STZ)-induced diabetic mice. After being orally administrated once a day for 24 days, CPF (300 mg/kg), CPP (180 mg/kg), or CPF+CPP (300 mg/kg CPF + 180 mg/kg CPP) treatment reversed STZ-induced body weight and muscle mass losses. The glucose tolerance tests and insulin tolerance tests suggested that CPF, CPP, and CPF+CPP showed anti-hyperglycemic effect in STZ-induced diabetic mice. Furthermore, CPF enhances glucose-stimulated insulin secretion in MIN6 cells and insulin-stimulated glucose uptake in C2C12 myotubes. CPF and CPP suppressed inflammatory cytokine levels in STZ-induced diabetic mice. Additionally, CPF and CPP improved STZ-induced diabetic nephropathy assessed by H&E staining, blood urea nitrogen content, and urine creatinine level. The molecular networking and Emperor analysis results indicated that CPF showed potential anti-hyperglycemic effects, and HPLC–MS/MS analysis indicated that CPF contains 3 phenolic acids and 9 flavonoids. In contrast, CPT (650 mg/kg) and CPC (300 mg/kg CPF + 180 mg/kg CPP + 650 mg/kg CPT) did not show anti-hyperglycemic effect. Taken together, polysaccharides and flavonoids are responsible for the anti-hyperglycemic effect of *C. paliurus* leaves, and the clinical application of *C. paliurus* need to be refined.

## 1. Introduction

Diabetes mellitus is increasing at an alarming rate worldwide, especially in developing countries [1,2]. Type 2 diabetes is characterized by hyperglycemia and insulin deficiency [3,4,5]. Long-term hyperglycemia causes damage, dysfunction, and failure of various organs, especially the eyes, kidneys, nerves, heart, and blood vessels [6]. Hyperglycemia-associated muscle mass and functional loss appear in the very early stage of diabetes [7]. A recent study showed high blood glucose decelerates WWP1-associated ubiquitous degradation of the transcription factor KLF15, resulting in muscle atrophy [8]. Diabetic nephropathy is a major complication of diabetes; poorly controlled diabetes causes damage to blood vessel clusters in the kidneys, leading to kidney damage and high blood pressure [9]. Since the clinical application of anti-diabetic drugs, including sulfonylureas, metformin, and thiazolidinediones, always have unpleasant side effects such as nausea, weight gain, headache, and dizziness [10,11], more and more interest has been directed towards natural products for the discovery of anti-hyperglycemic agents.

*Cyclocarya paliurus* (Batal.) Iljinsk (Juglandaceae) is native to eastern and central China, with the Chinese name “Qing Qian Liu” or “sweet tea tree” [12]. The leaves of *C. paliurus* have been widely used as ethnic medicine or herbal tea to treat diabetes in China. In recent years, the extracts of *C. paliurus* leaves were revealed to reduce blood glucose and improve insulin sensitivity on different diabetic models [13,14,15]. Polysaccharides from *C. paliurus* leaves were reported to display anti-diabetic activity in alloxan-induced mice [16]. Flavonoids isolated from *C. paliurus* leaves showed potential anti-diabetic activity in high-fat-diet-fed and streptozotocin (STZ)-stimulated mice; and the major constituents, including quercetin-3-*O*-glucuronide and kaempferol-3-*O*-glucuronide, are responsible for the anti-hyperglycemic activity [17]. Several triterpenoids isolated from *C. paliurus* leaves were found to enhance insulin-stimulated glucose uptake in both C2C12 myotubes and 3T3-L1 adipocytes [18]. However, the anti-hyperglycemic potential of different fractions from *C. paliurus* leaves has never been compared side-by-side on the same model. The chemical principles responsible for the anti-hyperglycemic effect of *C. paliurus* leaves remain controversial. Herein, different fractions were prepared from *C. paliurus* leaves. Total flavonoids (CPF) were purified using polyamide resin and D101 macroporous adsorption resin, characterized by UPLC-Q-TOF-MS [19]. *C. paliurus* polysaccharides (CPP) were obtained by the water-extraction and alcohol-precipitation method [20]. Total triterpenoids (CPT) were extracted by ethanol and then purified using AB-8 macroporous resin and a gradient ethanol elution [21]. Their anti-hyperglycemic effects were evaluated on STZ-induced mice using glucose tolerance tests, insulin tolerance tests, and homeostasis model assessment of basal insulin resistance. Glucose-stimulated insulin secretion in MIN6 cells was performed to evaluate the protective effect of the fractions on pancreatic β-cell function. Diabetic nephropathy was evaluated by H&E staining and biochemical tests. Finally, molecular networking and Emperor analysis of the LC–MS/MS data provided chemical profiles of the active fraction. The purpose of the current study is to uncover the chemical constituents responsible for the anti-hyperglycemic effect of *C. paliurus* and further guide the clinical application of this herbal material.

## 2. Results

### 2.1. CPF and CPP Reverse Body Weight and Muscle Weight Losses in STZ-Induced Diabetic Mice

**Figure 1:**
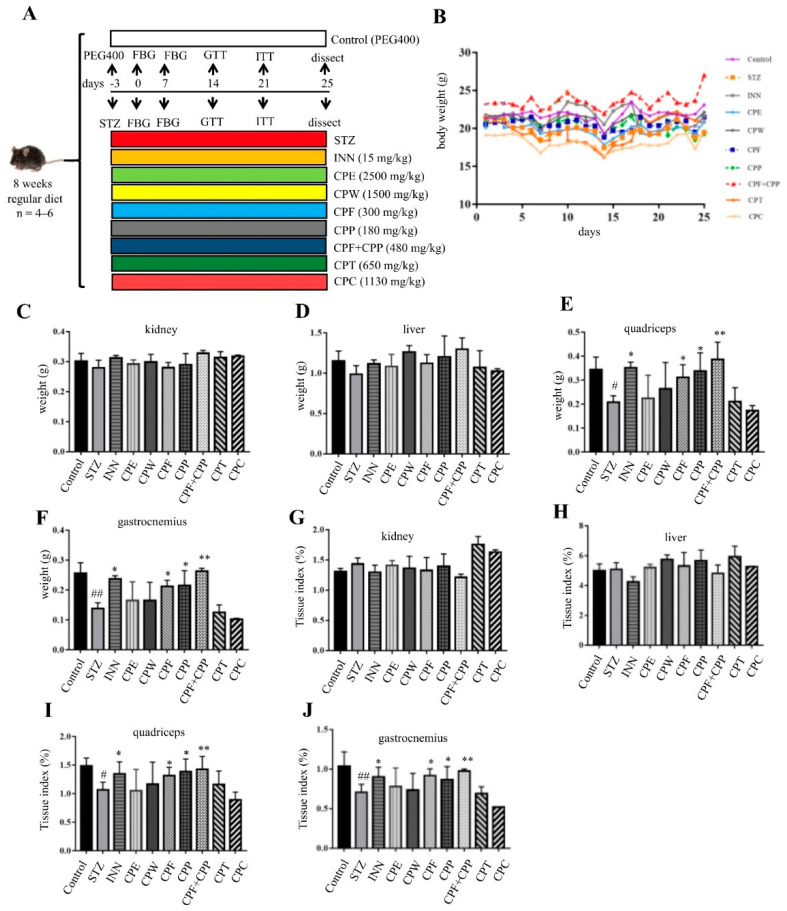
CPF (flavonoids fraction from *C. paliurus* leaves) and CPP (polysaccharide fraction from *C. paliurus* leaves) reverse body weight and muscle weight losses in STZ (streptozotocin)-induced diabetic mice. (**A**) The experiment procedure for STZ-induced diabetic mice. The male C57BL/6J mice were ip administrated with 150 mg/kg STZ, and then the mice were orally administrated with or without *C. paliurus* fractions once a day for 24 days. INN (glibenclamide, 15 mg/kg); CPE (ethanol extract from *C. paliurus* leaves, 2500 mg/kg); CPW (water extract from *C. paliurus* leaves, 1500 mg/kg); CPF (300 mg/kg); CPP (180 mg/kg); CPF+CPP (flavonoids and polysaccharide fractions from *C. paliurus* leaves, 480 mg/kg); CPT (triterpenoids fraction from *C. paliurus* leaves, 650 mg/kg); CPC (flavonoids, polysaccharide, and triterpenoids fractions from *C. paliurus* leaves, 1130 mg/kg). (**B**) Bodyweight of mice. (**C**–**F**) Kidneys, liver, quadriceps, and gastrocnemius weights of each group. (**G**–**J**) Tissue indexes of kidneys, liver, quadriceps, and gastrocnemius. *n* = 6. # *p* < 0.05, ## *p* < 0.01, STZ vs. control, * *p* < 0.05, ** *p* < 0.01, STZ vs. treatment.

To evaluate the anti-hyperglycemic effect of the refined fractions from *C. paliurus* leaves, STZ-induced mice were recruited (Figure 1A). INN (15 mg/kg) was used as a positive control [22]. The body weight from the STZ group of mice was significantly decreased in comparison to that of the control group (Figure 1B); 25 days treatment of CPC or CPT slightly reversed body weight loss compared to the STZ group, and treatment of CPF, CPP, or CPF+CPP reversed STZ-induced body weight loss (Figure 1B). Next, the weights of kidney, liver, quadriceps, and gastrocnemius from each group were compared, and the corresponding tissue indexes were calculated. The weights of quadriceps and gastrocnemius from STZ group were significantly reduced, but not kidney or liver, when compared with those of the control group (Figure 1C–F). The weight losses of quadriceps and gastrocnemius from CPP, CPF, and CPP + CPF groups were obviously lower than those of the STZ group, which were comparable with those of the control group (Figure 1E,F). The weights of quadriceps and gastrocnemius from CPT and CPC groups were similar to those of the STZ group (Figure 1E,F). The same trend was observed for the tissue indexes of quadriceps and gastrocnemius (Figure 1I,J). On the other hand, the weights or indexes of liver and kidneys were unchanged in all treatments (Figure 1C,D,G,H). The above results suggest that CPF, CPP, or CPF + CPP treatment reversed STZ-induced body weight and muscle mass losses.

### 2.2. CPF and CPP Improve Insulin Sensitivity in STZ-Induced Diabetic Mice

**Figure 2:**
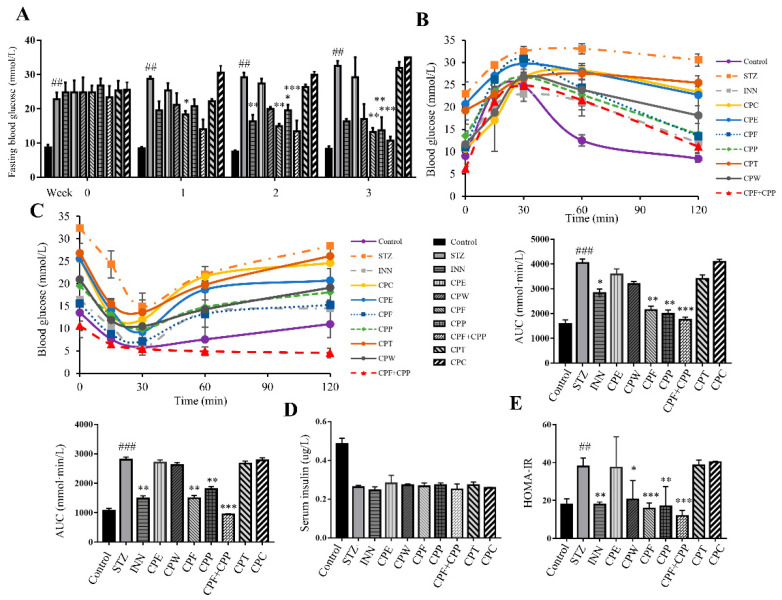
CPF (flavonoids fraction from *C. paliurus* leaves) and CPP (polysaccharide fraction from *C. paliurus* leaves) improve insulin sensitivity in STZ (streptozotocin)-induced diabetic mice. (**A**) Fasting blood glucose levels of all groups. (**B**) Glucose tolerance test was performed after 2-week CP fractions treatment. AUC of each group was calculated. (**C**) Insulin tolerance test was performed after 3-week CP fractions treatment. AUC (area under curve) of each group was calculated. (**D**) The serum insulin levels were determined after 18 h fasting. (**E**) The homeostasis model assessment of basal insulin resistance (HOMA-IR). *n* = 6. ## *p* < 0.01, ### *p* < 0.001, STZ vs. control, * *p* < 0.05, ** *p* < 0.01, ***p < 0.001, STZ vs. treatment.

In STZ-treated mice, the fasting blood glucose (FBG) levels were significantly increased in comparison to those in the control group (Figure 2A), indicating impaired β-cell function. After one week’s treatment, the FBG in CPF group was significantly reduced when compared with STZ group (Figure 2A). After three weeks’ treatment, the FBGs in CPF, CPP, CPF+CPP, and INN groups were significantly reduced when compared with STZ group; and the FBG in CPW group was slightly decreased, but not the CPE, CPT, or CPC group (Figure 2A). During GTT, the glucose clearance rates in the STZ, CPT, and CPC groups were greatly interrupted when compared with that of the control group, indicating an impaired pancreatic β-cell function in these groups of mice (Figure 2B). The CPF, CPP, and CPF+CPP treatment improved glucose disposal rate, comparable with that of INN-treated mice (Figure 2B). During ITT, the blood glucose levels of CPF, CPP, and CPF + CPP treated mice were significantly reduced under insulin stimulation compared to the STZ group (Figure 2C). After STZ treatment, the insulin levels of all groups were decreased, and the serum insulin level did not change in each treatment, indicating that these treatments did not rescue β-cell function (Figure 2D). HOMA-IR calculation results indicate that CPF, CPP, CPF + CPP, and INN treatment groups markedly attenuated insulin resistance compared to the STZ-induced diabetic mice group (Figure 2E). Taking the above results together, CPF, CPP, and CPF + CPP treatment showed anti-hyperglycemic effect in STZ-induced mice.

### 2.3. CPF Enhances Glucose-Stimulated Insulin Secretion in MIN6 Cells and Insulin-Stimulated Glucose Uptake in C2C12 Myotubes

**Figure 3:**
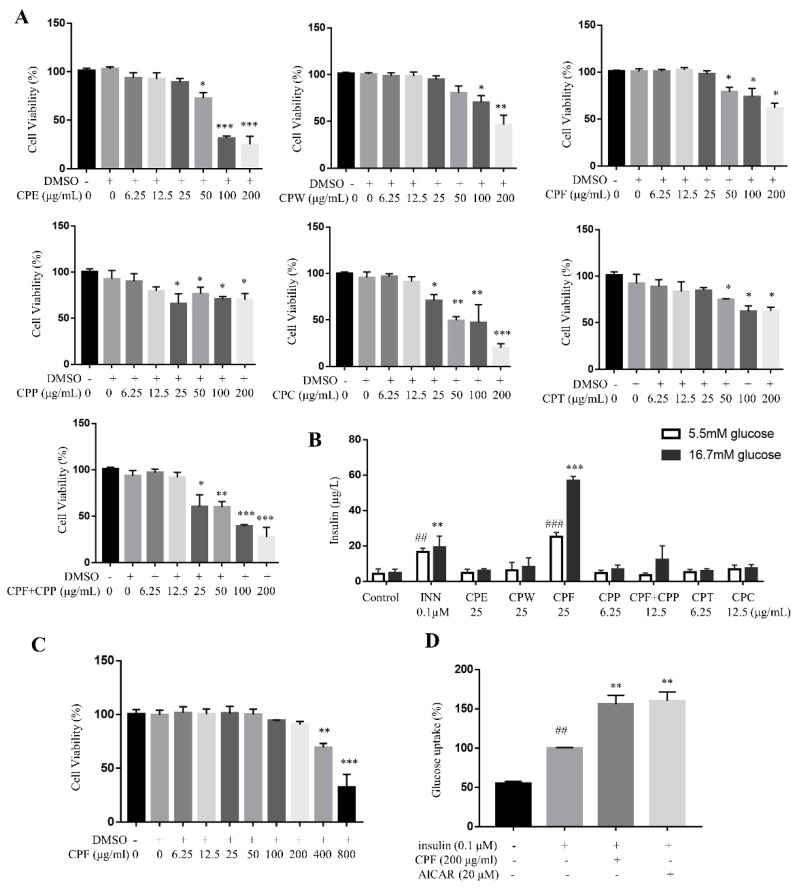
CPF (flavonoids fraction from *C. paliurus* leaves) enhanced glucose-stimulated insulin secretion in MIN6 cells and insulin-stimulated glucose uptake in C2C12 myotubes. (**A**) Cytotoxicity of different CP fractions on MIN6 cells, determined by MTT assay. *n* = 6, * *p* < 0.05, ** *p* < 0.01, *** *p* < 0.001, vs. DMSO. (**B**) CPF promoted glucose-stimulated insulin secretion in MIN6 cells. *n* = 6, ## *p* < 0.01, ### *p* < 0.001, vs. control cells in 5.5 mM glucose; ** *p* < 0.01, *** *p* < 0.001, vs. control cells in 16.7 mM glucose. (**C**) Cytotoxicity of CPF on C2C12 cells. (**D**) CPF promoted insulin-stimulated glucose uptake in C2C12 myotubes. *n* = 6, ##*p* < 0.01 insulin vs. control, ** *p* < 0.01, *** *p* < 0.001, CPF vs. insulin.

The flavonoids from *C. paliurus* leaves have been reported to improve insulin sensitivity [17,23]. Herein, we evaluated the effects of different CP fractions on glucose-induced insulin secretion in MIN6 cells. The MTT assay determined the maximum safe concentration of each fraction (Figure 3A). Only CPF (25 µg/mL) enhanced insulin secretion in both 5.5 mM- and 16.7 mM-stimulated MIN6 cells, which was comparable with INN-treated cells (Figure 3B). CPF enhances insulin secretion in normal pancreatic β cells.

The anti-hyperglycemic effect of CPF might be due to enhanced insulin sensitivity of skeletal muscle. Firstly, the cytotoxicity of CPF on C2C12 myotubes was evaluated to determine the maximum safe dosage. The results show CPF did not show obvious cytotoxicity on C2C12 myotubes up to 200 μg/mL (Figure 3C). Next, CPF was found to enhance insulin-stimulated glucose uptake on C2C12 myotubes under the dosage of 200 μg/mL, comparable with the positive control AICAR (5-aminoimidazole-4-carboxamide1-β-D-ribofuranoside, Figure 3D). Thus, CPF enhances insulin sensitivity on C2C12 myotubes.

### 2.4. CPF and CPP Suppressed Inflammatory Cytokine Levels in STZ-Induced Diabetic Mice

**Figure 4:**
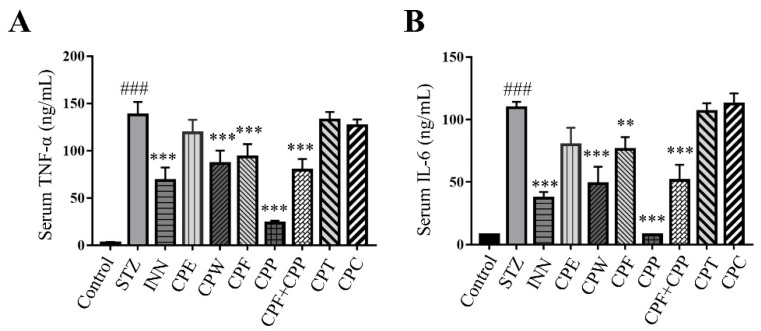
CPF (flavonoids fraction from *C. paliurus* leaves) and CPP (polysaccharide fraction from *C. paliurus* leaves) suppressed inflammatory cytokine levels in STZ (streptozotocin)-induced diabetic mice. (**A**) The levels of serum tumor necrosis factor-*α* (TNF-*α*) were determined by ELISA. (**B**) The levels of serum interleukin-6 (IL-6) were determined by ELISA. *n* = 6, ### *p* < 0.001, STZ vs. control, ** *p* < 0.01, *** *p* < 0.001, STZ vs. treatment.

Inflammation can be triggered by structural components of gut bacteria, resulting in a cascade of inflammatory pathways involving interleukins and other cytokines [24]. Previous studies showed that pro-inflammatory cytokines interleukin-6 (IL-6) and tumor necrosis factor-α (TNF-α) were associated with microbiota imbalance in diabetic mice [25,26]. Thus, the anti-hyperglycemic effect of CPF and CPP might be due to microbiota modulation. As shown in Figure 4A,B, the serum TNF-α and IL-6 levels were increased in STZ-treated mice, and the CPF, CPP, and CPF + CPP treatment significantly decreased the serum TNF-α and IL-6 levels compared to the STZ group. These results indicate that CPF and CPP possess anti-inflammation effect on STZ-induced mice.

### 2.5. CPF and CPP Improved Nephropathy in STZ-Induced Diabetic Mice

**Figure 5:**
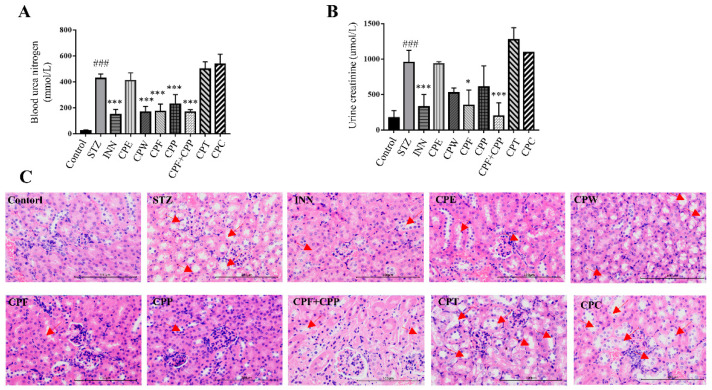
CPF (flavonoids fraction from *C. paliurus* leaves) and CPP (polysaccharide fraction from *C. paliurus* leaves) improved nephropathy in STZ (streptozotocin)-induced diabetic mice (**A**) The blood urea nitrogen levels of all CP treatment group. (**B**) The urine creatinine levels of all CP treatment groups. (**C**) Representative H&E staining of the kidney. Scale bar = 100 μm. The exfoliated renal tubes are indicated by the red arrows. *n* = 6, ### *p* < 0.001, STZ vs. control, * *p* < 0.05, *** *p* < 0.001, STZ vs. treatment.

Diabetic nephropathy is a severe complication of diabetes, due to long-term high blood glucose levels [27,28]. Diabetic nephropathy further progresses to kidney failure, a life-threatening condition [29]. To study the influence of C. paliurus fractions on diabetic nephropathy, blood urea nitrogen and urine creatinine levels were tested. The blood urea nitrogen and urine creatinine levels were greatly elevated in STZ group and significantly reversed in CPW, CPF, CPP, CPF + CPP, and INN groups (Figure 5A,B). CPE, CPT, and CPC treatment did not change the blood urea nitrogen or urine creatinine levels (Figure 5A,B). In H&E staining of kidney, it appeared in the STZ group that the epithelial cells of renal tubules were exfoliated, the basement membrane of renal tubules was exposed, the renal tubules were compensatory dilated and incised, the glomeruli were hypertrophic, and the basement membrane became thicker; these symptoms were reversed in CPF, CPP, and CPF + CPP treatment groups (Figure 5C). The kidney damage was even worse in CPT and CPC treatment groups compared with the STZ group (Figure 5C). Taken together, CPF and CPP protect against STZ-induced diabetic nephropathy.

### 2.6. The Bioinformatics Predict Results Indicate CPF with Potential Anti-Diabetic Effects

**Figure 6:**
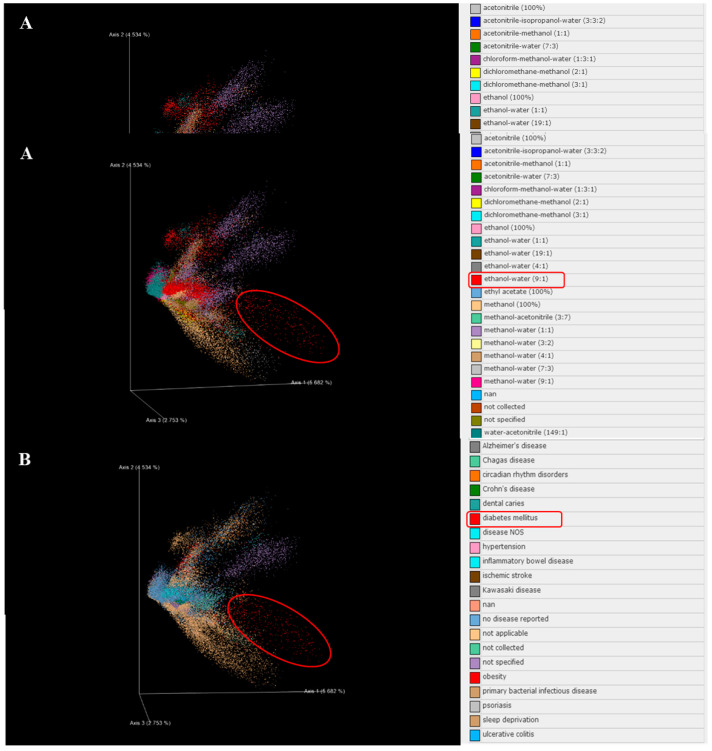
CPF (flavonoids fraction from *C. paliurus* leaves) ion fragments were collected from diverse solvent sources and showed potential anti-diabetic effects using bioinformatics prediction. (**A**) LC–MS^2^ (liquid chromatography–mass spectrometry) to GNPS (Global Natural Product Social Molecular Networking) and Emperor analysis results show diverse solvent sources. (**B**) LC–MS^2^ to GNPS and Emperor analysis results show potential anti-diabetic effect.

Based on the results from liquid chromatography–mass spectrometry (LC–MS) and GNPS and Emperor analysis, the ion fragments of CPF from diverse solvents were pooled together, such as 100% acetonitrile, acetonitrile–water (7:3), acetonitrile–methanol (1:1), 100% ethanol, 90% ethanol, and 100% methanol; some ion fragments were not collected or specified based on the databases (Figure 6A). The predicted ion fragments with potential anti-diabetic activity are highlighted with red circles (Figure 6B). The results indicate that the fragments in CPF with potential anti-diabetic activity were mainly distributed in 90% ethanol (highlighted with a red circle).

### 2.7. Characterization of Chemical Constituents in CPE, CPF, CPP, and CPT

Before data processing, an in-house formula database involving compound name, molecular formula, chemical structure, accurate mass, and related product ions of the compounds in *Cyclocarya paliurus* was established by searching from databases such as Reaxys (https://www.reaxys.com/ accessed on 8 March 2020), PubMed (http://www.ncbi.nlm.nih.gov/pubmed accessed on 8 March 2020), and CNKI (http://www.cnki.net accessed on 8 March 2020).

**Figure 7:**
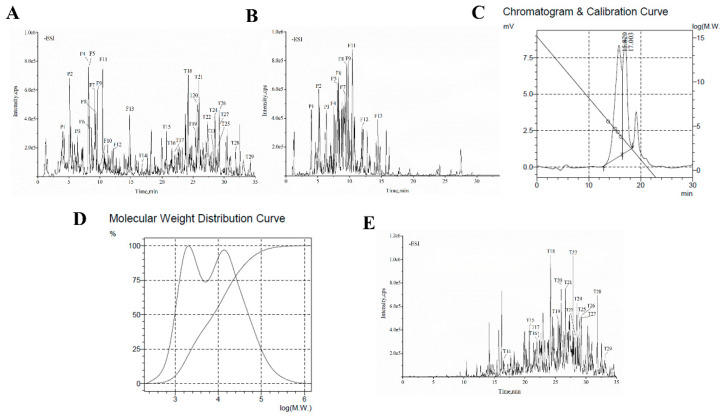
(**A**) Total ion chromatograms of CPE (ethanol extract from *C. paliurus* leaves, (**B**) CPF (flavonoids fraction from *C. paliurus* leaves), and (**C**) CPT (triterpenoids fraction from *C. paliurus* leaves), in negative ion mode. (**D**) Chromatogram and calibration curve of CPP. (**E**) Molecular weight distribution curve of CPP.

The typical total ion chromatography (TIC) profiles of CPE in the negative ion mode were presented in Figure 7A. A total of 29 compounds were identified, including 3 phenolic acids, 10 flavonoids, and 16 triterpenoids (Table 1). These compounds were 3-*O*-caffeoylquinic acid, 4-*O*-caffeoylquinic acid, catechin, isoquercetin, quercetin-3-*O*-glucuronide, kaempferol-3-*O*-galactoside, kaempferol-3-*O*-glucuronide, kaempferol-3-*O*-glucoside, quercetin-3-*O*-rhamnoside, myricetin, kaempferol-3-*O*-rhamnoside, quercetin, kaempferol, 3β,19α,23-trihydroxy-1-oxo-olean-12-en-28-oic acid, arjunolic acid, cyclocaric acid B, asiatic acid, pterocaryoside B, cyclocarioside I, cyclocarioside K, pterocaryoside A, cyclocariol C, (20*S*,24*R*)-20,24-epoxy-25-hydroxy-12*β*-(α-l-arabinopyranosyloxy)-3,4-seco-dammara-4-(28)-en-3-oic acid, cyclocarioside B, hederagenin, cyclocarioside H, cyclocarioside X, cypaliuruside E, and ursolic acid [17,30,31,32,33,34,35,36,37,38,39,40].

The TIC profiles of CPF in the negative ion mode were presented in Figure 7B. A total of 12 compounds were identified, including 3 phenolic acids and 9 flavonoids (Table 2). They were 3-*O*-caffeoylquinic acid, 4-*O*-caffeoylquinic acid, catechin, isoquercetin, quercetin-3-*O*-glucuronide, kaempferol-3-*O*-galactoside, kaempferol-3-*O*-glucuronide, kaempferol-3-*O*-glucoside, quercetin-3-*O*-rhamnoside, kaempferol-3-*O*-rhamnoside, quercetin, and kaempferol.

The GPC analysis result for CPP is shown in Figure 7C,D. The molecular weights of CPP were in the range of 30,000–50,000 Da (Figure 7D). The *Mn*, *Mw*, and *Mz* of the peak were 1.4844 × 10^4^, 3.6649 × 10^4^, and 1.31810 × 10^5^, respectively.

The TIC profiles of CPT in the negative ion mode are presented in Figure 7E. A total of 16 triterpenoids were identified (Table 3), including 3β,19α,23-trihydrox1-oxo-olean-12-en-28-oic acid, arjunolic acid, cyclocaric acid B, asiatic acid, pterocaryoside B, cyclocarioside I, cyclocarioside K, pterocaryoside A, cyclocariol C, (20S,24R)-20,24-epoxy-25-hydroxy-12β-(α-L-arabinopyranosyloxy)-3,4-seco-dammara-4(28)-en-3-oic acid, cyclocarioside B, hederagenin, cyclocarioside H, cyclocarioside X, cypaliuruside E, and ursolic acid.

### 2.8. The Molecular Networking for CPF Using GNPS Analysis

**Figure 8:**
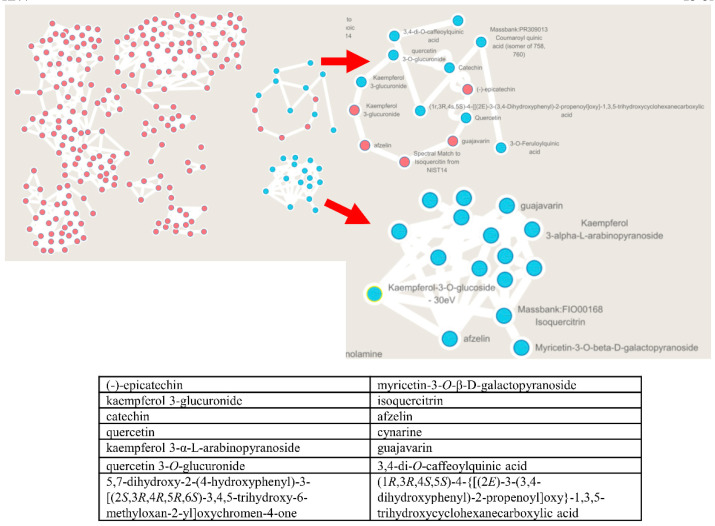
The molecular networking for CPF (flavonoids fraction from *C. paliurus* leaves) using GNPS (Global Natural Product Social Molecular Networking) analysis.

Public spectral libraries facilitate the dereplication of known molecules, and molecular networks allow annotation propagation of unknown related molecules. The aggregation of reference compounds in different clusters was based on different structural features. Compounds that were annotated as analogs were represented with a circular-colored node. Many flavonoids were identified from CPF. Some of them were reported to have anti-hyperglycemic effect, including epicatechin, myricetin-3-*O*-*β*-D-galactopyranoside, isoquercitrin, kaempferol 3-glucuronide, catechin, afzelin, quercetin, cynarine, kaempferol 3-*α*-L-arabinopyranoside, guajavarin, and quercetin 3-*O*-glucuronide, while other flavonoids’ pharmacological activities remained unknown, such as 3,4-di-*O*-caffeoylquinic acid and 5,7-dihydroxy-2-(4-hydroxyphenyl)-3-[(2*S*,3*R*,4*R*,5*R*,6*S*)-3,4,5-trihydroxy-6-methyloxan-2-yl]oxychromen-4-one,(1*R*,3*R*,4*S*,5*S*)-4-{[(2*E*)-3-(3,4-dihydroxyphenyl)-2-propenoyl]oxy}-1,3,5-trihydroxycyclohexanecarboxylic acid (Figure 8). Some previous undescribed compounds were predicted according to the analysis results. These results indicate that diverse flavonoids exist in CPF, which might contribute to the anti-diabetic property of CPF.

## 3. Discussion

*C. paliurus* is named as “sweet tea tree” in China because of the flavor of its leaves, which have been used as an herbal tea to treat hyperglycemia and obesity [42]. *C. paliurus* is enriched with flavonoids, triterpenoids, and polysaccharides. So far, over 200 compounds have been isolated and identified from *C. paliurus* [43], including 27 polysaccharides [44], 137 flavonoids [45], and 43 triterpenoids [18,46]. These isolates showed various pharmacological activities including anti-inflammation, anti-oxidation, anti-microbial, anti-cancer, and anti-diabetes activities [47,48,49]. Different extracts from *C. paliurus* were reported with anti-diabetic potential on different animal models [17,23,24,48,50]. Until now, no random-controlled clinical trial of *C. paliurus* has been reported. More studies are needed to identify potential therapeutic targets and chemical principles of *C. paliurus*, and clinical trials are necessary to verify the anti-hyperglycemic effect of *C. paliurus*.

The polysaccharides from *C. paliurus* leaves have attracted much research attention in search of an anti-hyperglycemic principle. Around 20 polysaccharides were isolated from *C. paliurus*, and their monosaccharide compositions were identified [47]. The molecular weights and compositions of polysaccharides isolated by different methods varied greatly. Based on the GPC results, the molecular weights of CPP were in the range of 30,000–50,000 Da. Considering polysaccharides could hardly pass through the gastrointestinal tract intact and enter into circulation, gut microbiota and their fermentative products, short chain fatty acids, are thought to medicate the physiological function of polysaccharides. Polysaccharides from *C. paliurus* leaves were found to reduce blood glucose levels and improve glucose tolerance and serum lipid parameters in high-fat-diet-induced diabetic mice through modulating gut microbiota balance and short-chain fatty acids content [21]. In high-fat-diet-induced diabetic rats, polysaccharides from *C. paliurus* leaves markedly attenuated the symptoms of diabetes, inhibited the protein expression of Bax, increased the expression of Bcl-2 in the pancreas, normalized hormones secretion, and alleviated the levels of inflammatory mediators, which contributed to the regeneration of pancreatic β-cell and attenuation of insulin resistance [24]. Herein, we found CPP reverses body weight and muscle weight losses, ameliorates inflammatory responses, and lowers blood glucose in STZ-induced mice. This evidence suggests the polysaccharides from *C. paliurus* could partially contribute to the anti-hyperglycemic effect, through regulating gut microbiota balance, protecting gut epithelial barrier, and ameliorating inflammatory responses in the hosts. Whereas the CPP was not deproteinized in the isolation procedure, and a few flavonoids were identified in the CPP fraction, including quercetin 3-*O*-glucuronide, afzelin, and kaempferol 3-glucuronide, the bioactivity of CPC might partially be contributed to by proteins and/or flavonoids. A refined total polysaccharides fraction should be prepared and re-evaluated in the future.

Flavonoids are the major constituents of *C. paliurus* leaves and contribute to various pharmacological activities of *C. paliurus*, including anti-diabetic effect. The major flavonoids, including quercetin-3-*O*-glucuronide, kaempferol-3-*O*-glucuronide, kaempferol-7-*O*-α-l-rhamnoside, kaempferol, and quercetin, were identified from *C. paliurus* leaves with potent anti-oxidative activity [36]. Quercetin was reported to activate insulin receptor and glucose transporter 4, which in turn elevate glucose uptake in Caco-2E, Caco-2, C2C12 murine skeletal myoblast, and H4IIE murine hepatocytes [51,52]. The anti-hyperglycemic effect of kaempferol and quercetin glycosides from *C. paliurus* was determined on high-fat-diet-fed male C57BL/6J mice [17,53]. CPF treatment obviously reduced blood glucose levels in STZ-induced diabetic mice, which might be due to its effect of enhancing glucose uptake in skeletal muscle. CPF could enhance glucose-stimulated insulin secretion on normal MIN6 cells but did not change serum insulin level in STZ-induced mice, which suggested CPF might promote pancreatic β-cell function in normal objects. Additionally, CPF administration greatly decreased the levels of TNF-α and IL-6, indicating its anti-inflammatory activity. Pro-inflammatory cytokines can cause insulin resistance in adipose tissue, skeletal muscle, and liver by inhibiting insulin signal transduction [54,55]. The combination of CPP and CPF showed more pronounced anti-hyperglycemic effect in STZ-treated mice, which suggests CPP and CPF might function through different mechanisms and possess additive or synergic effects.

Although our previous study indicated several triterpenoids from *C. paliurus* leaves enhance insulin-stimulated glucose uptake on myotubes and adipocytes, the treatment of CPT and CPC did not show anti-diabetic effect in STZ-induced diabetic mice. This might be because triterpenoids cannot pass through the gastrointestinal tract and reach the target organs. Some improvements could be achieved, such as using drug-wrapping materials to improve intestinal absorption and the bioavailability of triterpenes, and the mode of administration can be changed from intragastric injection to intraperitoneal injection.

The clinically used anti-diabetic medicines mainly include insulin sensitizers, α-glucosidase inhibitors, and insulin-secreting agents. In recent years, glucagon-like peptide 1 receptor agonists, dipeptidyl peptidase-4 inhibitors, and sodium-glucose cotransporter-2 inhibitors have been successfully developed as anti-diabetic agents. Gut microbiota imbalance and, subsequently, systematic inflammation and metabolic disorders are positively correlated with insulin resistance, which has attracted more and more interest. Although many studies have indicated different extracts from *C. paliurus* possess anti-diabetic activity, refined fractions have never been compared on the same model. The dosage of each fraction was determined by the extraction rate, which was equal to the same amount of herbal material. Our study revealed for the first time that the polysaccharides and flavonoids from *C. paliurus* are responsible for its anti-hyperglycemic effect. This will benefit the clinical application of *C. paliurus* in the future. Bioinformatic tools including GNPS and Emperor were applied in the current study, which can help us better understand and predict bioactive principles from a complex extract and shorten the isolation and purification procedure.

We investigated the anti-hyperglycemic activity of refined fractions from *C. paliurus* on a high-dosage injection of STZ-induced diabetic mice model, which is more likely a type 1 diabetic model. Alternatively, high-fat diet feeding plus low-dosage STZ injection could induce type 2 diabetic mellitus phenotypes, which is more suitable for evaluating the anti-hyperglycemic effect of *C. paliurus*. Second, the glucose-stimulated insulin secretion in isolated islets of CP-fraction-treated mice should be performed, it can help us better understand the role of CP fraction in rescuing β-cell function. Additionally, some of the predicted compounds with potential anti-diabetic function have not been verified, and the systemic evaluation of these compounds should be carried out in the future.

## 4. Materials and Methods

### 4.1. Plant Material

The leaves of *C. paliurus* (Batal.) Iljinsk were collected in Sangzhi County, Zhangjiajie City, Hunan Province, China, in March 2018, which were identified by Jian-Xia Mo from Zhejiang University. A voucher specimen (accession number CP-2018-I) was deposited in the Institute of Modern Chinese Medicine, Zhejiang University (Hangzhou, China).

### 4.2. Extraction and Preparation

**Figure 9:**
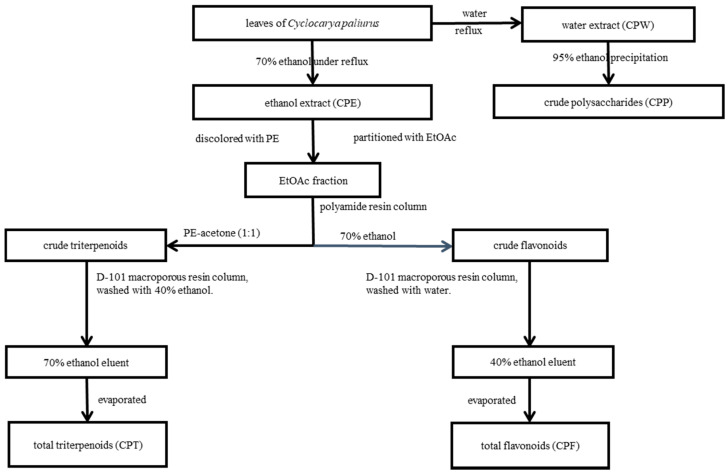
Extraction and purification procedures of *C. paliurus* leaves.

The extraction and purification procedures are shown in Figure 9. Air-dried leaves of *C. paliurus* (3 kg) were extracted with 70% ethanol (3 × 30 L) under reflux and evaporated to produce an ethanol extract (CPE, 534.0 g). The residue leaves were then extracted again with boiling distilled water (3 × 30 L), and a water extract (CPW, 220.9 g) was obtained under reduced pressure. A part of CPW was then dissolved with water and added with 5 parts of 95% ethanol. After sitting at room temperature overnight, the precipitate was collected by filtration and dried under vacuum to obtain the crude polysaccharides (CPP, 27.6 g). A part of CPE was suspended in water (1 L) and successively partitioned with petroleum ether (PE) and ethyl acetate (EtOAc) to obtain the EtOAc fraction. The EtOAc fraction (175.4 g) was first subjected to column chromatography over polyamide resin eluted with PE-acetone (1:1) to obtain the crude triterpenoids (87.6 g), followed by aqueous EtOH (70%) to obtain the crude flavonoids (45.8 g). Next, the crude triterpenoids were purified on a D-101 macroporous resin column eluted with aqueous EtOH (40%) to remove impurities. Then, 70% ethanol eluent was collected and subsequently evaporated under reduced pressure to yield the total triterpenoids (CPT, 40.2 g). Similarly, the crude flavonoids were dispersed in water and purified on a D-101 macroporous resin column. The water-soluble impurities were first removed with water, and 40% ethanol eluent was collected. The total flavonoids (CPF, 10.2 g) were obtained after removal of the solvent under reduced pressure.

### 4.3. Animals

All animal care and experimental procedures followed the guidelines and regulations approved by the Animal Ethical and Welfare Committee of University of Macau (No. UMARE-033-2017). Male C57BL/6J mice (8–10 weeks old) were obtained from the animal facility of the Faculty of Health Science, University of Macau (Macau, China). The mice were housed at 22 ± 1 °C with 12-h light–dark cycles and fed with a regular chow diet (Guangdong Medical Lab Animal Center, Guangzhou, Guangdong, China) and water ad libitum under standard conditions (specific-pathogen-free) with air filtration.

### 4.4. STZ-Induced Diabetic Mice

The diabetes mice model was induced by intraperitoneal injection (i.p) with STZ (150 mg/kg) once. After 3 days, the mice with fasting blood glucose level between 11‒28 mmol/L were considered as diabetic mice. The diabetic mice were then randomly divided into nine groups, including STZ (model), CPE (ethanol extract from *C. paliurus* leaves), CPW (water extract from *C. paliurus* leaves), CPF (flavonoids fraction from *C. paliurus* leaves), CPP (polysaccharide fraction from *C. paliurus* leaves), CPT (triterpenoids fraction from *C. paliurus* leaves), CPF + CPP (flavonoids and polysaccharide fractions from *C. paliurus* leaves), CPC (flavonoids, polysaccharide, and triterpenoids fractions from *C. paliurus* leaves), and Glibenclamide (INN) used as a positive control [43,56,57,58]. The mice were orally administrated the same volume of 60% PEG400 solution (control and STZ), 15 mg/kg INN (INN), 2500 mg/kg CPE (CPE), 1500 mg/kg CPW (CPW), 300 mg/kg CPF (CPF), 180 mg/kg CPP (CPP), 650 mg/kg CPT (CPT), 300 mg/kg CPF + 180 mg/kg CPP (CPF + CPP), and 300 mg/kg CPF + 180 mg/kg CPP + 650 mg/kg CPT (CPC), respectively, once a day (Figure 2A). At the end of the experiment (day 25), the mice were dissected after carbon dioxide inhalation, and the kidneys, liver, and skeletal muscles (quadriceps and gastrocnemius) were collected.

### 4.5. Glucose Tolerance Tests (GTT) and Insulin Tolerance Tests (ITT)

GTT and ITT were performed at 2 and 3 weeks post-treatment, respectively, as described previously [59]. After 18 h fasting, the tail blood glucose was measured using the OneTouch Ultra blood glucose meter and LifeScan test strips. Then, the mice received an intraperitoneal injection of glucose solution (Sigma-Aldrich, St. Louis, MO, USA) at a dose of 1.5 g/kg body weight. The tail blood glucose was measured at 15, 30, 60, and 120 min after injections. For the ITT, the tail blood glucose concentration was measured after 6 h fasting. Mice then received an intraperitoneal injection of human insulin (Eli Lilly, Indianapolis, IN, USA) at a dose of 1.0 U/kg body weight. Tail blood glucose concentration was measured at 15, 30, 60, and 120 min after injections.

### 4.6. Blood Sample Collection and Preparation

About 0.5 mL blood was collected in heparin tube. Plasma was obtained after a 3000 rpm centrifuge for 30 min at room temperature. Plasma insulin was determined using the Insulin assay kit (Sigma-Aldrich, St. Louis, MO, USA) according to the manufacturer’s instructions.

### 4.7. Homeostasis Model Assessment of Basal Insulin Resistance

The homeostasis model assessment of basal insulin resistance (HOMA-IR) index was calculated as follows to assess insulin resistance: fasting serum glucose × fasting serum insulin/22.5. Lower HOMA-IR values indicated greater insulin sensitivity, whereas higher HOMA-IR values indicated insulin resistance.

### 4.8. Cell Viability

Cell viability was determined by 3-(4,5-dimethylthiazol-2-yl)-2,5-diphenyltetrazolium bromide (MTT, Sigma-Aldrich, St. Louis, MO, USA) assay. C2C12 cells were seeded in 96-well plates at a density of 1 × 10^4^ cells per well. The fully differentiated myotubes were treated with the indicated concentrations of CPF for 24 h. Then, cell viability was determined by incubation with DMEM containing MTT (1 mg/mL) for 4 h, followed by dissolving the formazan crystals with 100 μL dimethyl sulfoxide (DMSO). The absorbance at 570 nm was measured by a SpectraMax M5 microplate reader (Molecular Devices, CA, USA). The calculation equation for relative cell viability was as follows: cell viability (%) = (As − A0)/(Ac − A0) × 100%, where As, A0, and Ac were the absorptions of the test sample, blank control, and negative control (DMSO), respectively.

### 4.9. Glucose-Stimulated Insulin Secretion

MIN6 cells were cultured in Dulbecco’s modified eagle medium (DMEM, Gibco, Carlsbad, CA, USA) supplemented with 10% fetal bovine serum (FBS, Gibco, Carlsbad, CA, USA) and 1% penicillin–streptomycin (P/S, Gibco, Carlsbad, CA, USA), in a humidified incubator with 5% CO_2_ at 37 °C. The experiments were performed between passages 16 and 24 [60]. The cells were cultured in 24-well plates with high-glucose DMEM (25 mM) and then treated with CP fractions for 24 h. INN (glibenclamide, 0.1 µM, Sigma–Aldrich, St. Louis, MO, USA) was used as a positive control [61]. Subsequently, the cells were washed twice with Krebs–Ringer bicarbonate buffer (KRBB: CaCl_2_ 2.5 mM; KCl 4.7 mM; KH_2_PO_4_ 1.2 mM; MgCl_2_ 1.2 mM; NaCl 120 mM; HEPES 10 mM; NaHCO_3_ 25 mM; pH = 7.4) and incubated with KRBB with 3 mM glucose for 30 min. The cells were washed twice with KRBB and then incubated with KRBB with 5.5 or 16.7 mM glucose for 1 h. The supernatants were collected, and insulin was measured by mouse insulin ELISA kit (Mercodia, Winston Salem, NC, USA).

### 4.10. C2C12 Cell Culture and Differentiation

Mouse C2C12 myoblasts were obtained from American Type Culture Collection (Manassas, VA, USA) and maintained in DMEM supplemented with 10% FBS and 1% P/S. C2C12 cells were differentiated as described previously [62]. In brief, C2C12 cells were grown to 90% confluence and incubated with DMEM containing 2% heat-inactivated horse serum (Gibco, Carlsbad, CA, USA) and 1% P/S for 6 days. Media were refreshed every other day. The fully differentiated myotubes were used for the following experiments.

### 4.11. Insulin-Stimulated Glucose Uptake

Insulin-stimulated glucose uptake was performed as described previously [63]. C2C12 myotubes were treated with different concentrations of the total flavonoids (CPF) for 24 h. Then, cells were washed with Krebs–Ringer phosphate (KRP) buffer (20 mmol/L HEPES, 137 mmol/L NaCl, 4.7 mmol/L KCl, 1.2 mmol/L MgSO_4_, 1.2 mmol/L KH_2_PO_4_, 2.5 mmol/L CaCl_2_, and 2 mmol/L pyruvate; pH 7.4) and incubated in KRP buffer with 0.2% bovine serum albumin (BSA, Sigma-Aldrich, St. Louis, MO, USA) for 3 h. To stimulate glucose uptake, cells were incubated with KRP buffer containing 0.1 μmol/L insulin (Sigma-Aldrich, St. Louis, MO, USA) for another 30 min. After being washed with KRP buffer once, cells were incubated in KRP containing 100 μmol/L 2-(*N*-(7-nitrobenzene-2-oxa-1,3-diazol-4-yl)amino)-2-deoxyglucose (2-NBDG, Sigma-Aldrich, St. Louis, MO, USA) for 30 min. The intracellular amount of 2-NBDG was measured at an excitation wavelength of 475 nm and an emission wavelength of 550 nm. Glucose uptake was further normalized by protein content.

### 4.12. Determination of Cytokines

The cytokines in mice serum were determined by using commercial ELISA kits (Neobioscience Technology Co., Ltd., Shenzhen, China), following the manufacturer’s instructions.

### 4.13. H&E Staining of Kidney

Whole-mount histochemical analysis of the kidney was performed as previously described [64]. After fixation in 4% paraformaldehyde, the kidney samples were embedded in paraffin. A total of 5 μm sections were deparaffinized and rehydrated, followed by hematoxylin and eosin (H&E) staining.

### 4.14. Sample Preparation

The CPE powder (0.25 g) was dissolved by adding methanol in a 25 mL volumetric flask. The sample solution was centrifuged at 13,000 rpm for 10 min. The supernatant was stored at −4 °C before use. The CPF and CPT solution was prepared in the same way.

### 4.15. Emperor Analysis

Emperor is a tool for analyzing, visualizing, and understanding high-throughput microbial ecology data sets [65]. Due to its customized graphical user interface, it has never been easier to drill down into new data sets to illuminate patterns hidden in the data. Emperor can be integrated into any microbial ecological quantitative analysis (QIIME) or scikit bio compatible data set because its high-quality of modifications and customizations or sciKit biocompatible datasets. Throughout the lightweight data files and hardware-accelerated graphics, emperor is more and more often used in N-dimensional analyzing. [66].

### 4.16. Chromatography and MS Conditions

The samples were analyzed using a Waters UPLC system (Waters, Milford, MA, USA) equipped with a Waters XSelect^®^ HSS T3 column (4.6 mm × 250 mm, 5 μm). The sample injection volume was set at 10 μL. The optimal mobile phase consisted of acetonitrile solution (A) and 0.1% formic acid aqueous solution (B) at a flow rate of 1.0 mL/min at 30 °C. The solvent gradient was used as follows: 5–30% A at 0–10 min, 30–70% A at 10–30 min, 70–95% A at 30–40 min. The MS analysis was carried out by a Triple TOF 5600 plus mass spectrometer (AB SCIEX, Framingham, MA, USA) equipped with an ESI source (AB SCIEX, Framingham, MA, USA). The mass spectrometer was operated in both positive and negative ion mode. The following operating parameters were used: scan range, *m*/*z* 100–2000 for TOF-MS scan and *m*/*z* 50–2000 for TOF-MS/MS scan; ion spray voltage, +5.5 kV for positive ion mode and −4.5 kV for negative ion mode; ion source heater, 600 °C for positive ion mode and 550 °C for negative ion mode; nebulizing gas (Gas 1, Air), 50 psi; Tis gas (Gas 2, Air), 50 psi; curtain gas (CUR, N_2_), 30 psi; collision energy, 40 V with a collision energy spread of ± 20 V; maximum allowed error, ±5 ppm. The experiments were run with 250 ms accumulation time for TOF-MS and 100 ms accumulation time for TOF-MS/MS. The exact mass calibration was performed automatically before each analysis by employing the Automated Calibration Delivery System. The accurate mass and composition for the precursor ions and fragment ions were analyzed using the Peakview software (AB SCIEX, version 1.2.0.3, Framingham, MA, USA) integrated with the instrument.

### 4.17. Determination of Homogeneity and Molecular Weight for CPP

The molecular weight distribution of CPP was determined using gel permeation chromatography (GPC) method on a Shimadzu LC20 instrument equipped with a TSK GMPWXL gel filtration column (7.6 × 300 mm) and a differential refractive index (DRI) detector (Shimadzu RID-20A). CPP was dissolved in ultrapure water (5 mg/mL) and filtered through a 0.22 μm membrane prior to sample injection. Then, 20 μL of sample solution was injected in each run with water containing 0.1 N sodium nitrate and 0.06% sodium azide as the mobile phase at a flow rate of 0.6 mL/min in 35 ◦C. The molecular weight of polysaccharide sample was estimated by reference to the calibration curve made using dextran standards. The number-average molecular weight (*Mn*), weight-average molecular weight (*Mw*), Z-average molecular weight (*Mz*), and polydispersity were calculated using molecular weight calculation software connected to the GPC integration system, as indicated before [67].

### 4.18. Molecular Networking Workflow Description

A molecular network was created using an online workflow application (https://ccms-ucsd.github.io/GNPSDocumentation/ accessed on 25 March 2021) on the Global Natural Product Social Molecular Networking (GNPS) website (http://gnps.ucsd.edu accessed on 25 March 2021) [68]. The data were filtered by removing all MS/MS fragment ions within +/− 17 Da of the precursor *m/z*. MS/MS spectra were window-filtered by choosing only the top 6 fragment ions in the +/− 50 Da window throughout the spectrum. The precursor ion mass tolerance was set to 0.01 Da and an MS/MS fragment ion tolerance of 0.0075 Da. A network was then created in which edges were filtered to have a cosine score above 0.7 and more than 6 matched peaks. Further, edges between two nodes were kept in the network if and only if each of the nodes appeared in each other’s respective top 10 most similar nodes. Finally, the maximum size of a molecular family was set to 100, and the lowest-scoring edges were removed from molecular families until the molecular family size was below this threshold. The spectra in the network were then searched against GNPS’s spectral libraries. The library spectra were filtered in the same manner as the input data. All matches kept between network spectra and library spectra were required to have a score above 0.7 and at least 6 matched peaks.

### 4.19. Statistical Analysis

Data were analyzed using GraphPad Prism 7.0 (GraphPad Software, San Diego, CA, USA). All experimental data were expressed as mean ± S.D., and the sample size for each experiment corresponds to three biological replicates. Significant differences between groups were determined using a one-way analysis of variance (ANOVA) with Dunnet’s multiple comparisons test, considering *p* < 0.05 as significant differences. Where statistical significance is evaluated, the variance between groups is confirmed to be similar between comparison groups (control vs. experimental), and the statistical analysis is considered appropriate.

## 5. Conclusions

In summary, polysaccharides and flavonoids are responsible for the anti-diabetic effect of *C. paliurus* leaves, but not triterpenoids. For clinical application, the polysaccharides and flavonoids in *C. paliurus* leaves need to be enriched to enhance anti-hyperglycemic activity.

## Figures and Tables

**Table 1:** Identification of the chemical constituents in CPE by HPLC-ESI-Q-TOF-MS/MS.

No.	t_R_ (min)	Identification	Formula	Selected Ion	Measured (*m*/*z*)	Calculated (*m*/*z*)	Error (ppm)	MS^2^ (*m*/*z*)
P1	4.004	3-*O*-caffeoylquinic acid [41]	C_16_H_18_O_9_	[M−H]^−^	353.0884	353.0878	1.7	191.0556 (100) [M−H−CA+H_2_O]^−^
								179.0347 (40) [CA−H]^−^
								135.0450 (48) [CA−H−CO_2_]^−^
P2	5.084	4-*O*-caffeoylquinic acid [41]	C_16_H_18_O_9_	[M−H]^−^	353.0885	353.0878	2.0	191.0557 (100) [M−H−CA+H_2_O]^−^
P3	6.180	catechin [30]	C_15_H_14_O_6_	[M−H]^−^	289.0724	289.0718	2.2	289.0725 (100) [M−H]^−^
								271.0615 (8) [M−H−H_2_O]^−^
F4	8.094	isoquercetin [31,41]	C_21_H_20_O_12_	[M−H]^−^	463.0881	463.0882	−0.2	463.0910 (13) [M−H]^−^
								301.0347 (31) [M−H−glc]^−^
F5	8.145	quercetin-3-*O*-glucuronide [35,41]	C_21_H_18_O_13_	[M−H]^−^	477.0675	477.0675	0.1	477.0693 (13) [M−H]^−^
								301.0353 (100) [M−H−glcA]^−^
								283.0248 (10) [M−H−glcA−H_2_O]^−^
F6	8.872	kaempferol-3-*O*-galactoside [17,41]	C_21_H_20_O_11_	[M−H]^−^	447.0934	447.0933	0.3	447.0937 (56) [M−H]^−^
								285.0393 (31) [M−H−gal]^−^
F7	9.126	kaempferol-3-*O*-glucuronide [17,41]	C_21_H_18_O_12_	[M−H]^−^	461.0728	461.0725	0.5	461.0741 (5) [M−H]^−^
								285.0397 (100) [M−H−glcA]^−^
F8	9.198	kaempferol-3-*O*-glucoside [17,41]	C_21_H_20_O_11_	[M−H]^−^	447.0932	447.0933	−0.2	447.0926 (44) [M−H]^−^
								285.0390 (71) [M−H−glc]^−^
F9	9.297	quercetin-3-*O*-rhamnoside [17,41]	C_21_H_20_O_11_	[M−H]^−^	447.0928	447.0933	−1.1	447.0953 (19) [M−H]^−^
								301.0347 (56) [M−H−rha]^−^
F10	10.024	myricetin [34]	C_15_H_10_O_8_	[M−H]^−^	317.0303	317.0303	0.0	317.0299 (100) [M−H]^−^
								178.9975 (35) [M−H−C_7_H_6_O_3_]^−^
F11	10.396	kaempferol-3-*O*-rhamnoside [35,41]	C_21_H_20_O_10_	[M−H]^−^	431.0981	431.0984	−0.6	431.1008 (24) [M−H]^−^
								285.0401 (99) [M−H−rha]^−^
F12	12.071	quercetin [35]	C_15_H_10_O_7_	[M−H]^−^	301.0354	301.0354	0.1	301.0341 (100) [M−H]^−^
								273.0392 (18) [M−H−CO]^−^
F13	14.152	kaempferol [36]	C_15_H_10_O_6_	[M−H]^−^	285.0410	285.0405	1.9	285.0401 (100) [M−H]^−^
T14	16.778	3β,19α,23-trihydroxy-1-oxo-olean -12-en-28-oic acid	C_30_H_46_O_6_	[M−H]^−^	501.3226	501.3222	0.9	501.3275 (100) [M−H]^−^
								455.3210 (71) [M−H−FA]^−^
T15	20.616	arjunolic acid	C_30_H_48_O_5_	[M−H]^−^	487.3451	487.3429	4.5	487.3462 (100) [M−H]^−^
T16	21.990	cyclocaric acid B	C_30_H_46_O_5_	[M−H]^−^	485.3276	485.3272	0.7	485.3300 (100) [M−H]^−^
T17	22.128	asiatic acid	C_30_H_48_O_5_	[M−H]^−^	487.3424	487.3429	−1.0	487.3458 (100) [M−H]^−^
								469.3347 (10) [M−H−H_2_O]^−^
T18	24.206	pterocaryoside B [37]	C_35_H_58_O_9_	[M−H]^−^	621.4007	621.4008	−0.2	621.4078 (100) [M−H]^−^
								489.3624 (15) [M−H−ara]^−^
T19	25.341	cyclocarioside I	C_41_H_70_O_12_	[M−HCOO]^−^	799.4865	799.4849	2.0	799.4962 (19) [M+HCOO]^−^
								753.4893 (100) [M−H]^−^
								621.4482 (5) [M−H−ara]^−^
T20	25.613	cyclocarioside K	C_41_H_70_O_12_	[M−HCOO]^−^	799.4864	799.4849	1.8	799.4962 (19) [M+HCOO]^−^
								753.4893 (100) [M−H]^−^
T21	25.892	pterocaryoside A [37]	C_36_H_60_O_9_	[M−H]^−^	635.4166	635.4165	0.2	635.4249 (100) [M−H]^−^
								489.3639 (13) [M−H−qui]^−^
T22	28.175	cyclocariol C [38]	C_30_H_50_O_5_	[M−HCOO]^−^	535.3636	535.3640	−0.8	489.3615 (100) [M−H]^−^
T23	28.499	(20S,24R)-20,24-epoxy-25-hydroxy-12β -(α-L-arabinopyranosyloxy)-3,4-*seco*- dammara-4(28)-en-3-oic acid	C_35_H_58_O_9_	[M−H]^−^	621.4010	621.4008	0.3	621.4072 (100) [M−H]^−^
								489.3617 (20) [M−H−ara]^−^
T24	28.763	cyclocarioside B	C_42_H_72_O_12_	[M−HCOO]^−^	813.5020	813.5006	1.7	813.5129 (44) [M+HCOO]^−^
								767.5066 (100) [M−H]^−^
T25	29.008	hederagenin	C_30_H_48_O_4_	[M−H]^−^	471.3475	471.3480	−1.0	471.3493 (100) [M−H]^−^
								453.3401 (10) [M−H−H_2_O]^−^
T26	29.171	cyclocarioside H	C_43_H_72_O_13_	[M−HCOO]^−^	841.4969	841.4955	1.7	841.5088 (76) [M+HCOO]^−^
								795.5014 (100) [M−H]^−^
								753.4900 (57) [M−H−C_2_H_2_O]^−^
T27	29.245	cyclocarioside X [39]	C_37_H_62_O_9_	[M−H]^−^	649.4324	649.4321	0.4	649.4394 (100) [M−H]^−^
								517.3948 (12) [M−H−ara]^−^
T28	31.812	cypaliuruside E [40]	C_36_H_58_O_8_	[M−H]^−^	617.4058	617.4059	−0.2	617.4131 (100) [M−H]^−^
								471.3508 (12) [M−H−qui]^−^
T29	33.431	ursolic acid	C_30_H_48_O_3_	[M−H]^−^	455.3527	455.3531	−0.8	455.3580 (100) [M−H]^−^

glc: glucosyl; gal: galactosyl; rha: rhamnosyl; ara: arabinosyl; qui: quinovosyl; glcA: glucuronic acid; CA: caffeic acid; FA: formic acid; AcOH: acetic acid.

**Table 2:** Identification of the chemical constituents in CPF by HPLC-ESI-Q-TOF-MS/MS.

No.	t_R_ (min)	Identification	Formula	Selected Ion	Measured (*m*/*z*)	Calculated (*m*/*z*)	Error (ppm)	MS^2^ (*m*/*z*)
P1	3.938	3-*O*-caffeoylquinic acid [41]	C_16_H_18_O_9_	[M−H]^−^	353.0884	353.0878	1.7	191.0556 (100) [M−H−CA+H_2_O]^−^
								179.0347 (38) [CA−H]^−^
								135.0454 (41) [CA−H−CO_2_]^−^
P2	5.083	4-*O*-caffeoylquinic acid [41]	C_16_H_18_O_9_	[M−H]^−^	353.0885	353.0878	2.0	191.0554 (100) [M−H−CA+H_2_O]^−^
P3	6.146	catechin [30]	C_15_H_14_O_6_	[M−H]^−^	289.0725	289.0718	2.6	289.0711 (88) [M−H]^−^
								271.0600 (7) [M−H−H_2_O]^−^
F4	8.009	isoquercetin [31,41]	C_21_H_20_O_12_	[M−H]^−^	463.0888	463.0882	1.3	463.0919 (30) [M−H]^−^
								301.0358 (44) [M−H−glc]^−^
F5	8.125	quercetin-3-*O*-glucuronide [35,41]	C_21_H_18_O_13_	[M−H]^−^	477.0684	477.0675	2.0	477.0709 (11) [M−H]^−^
								301.0362 (100) [M−H−glcA]^−^
								283.0258 (7) [M−H−glcA−H_2_O]^−^
F6	8.841	kaempferol-3-*O*-galactoside [35,41]	C_21_H_20_O_11_	[M−H]^−^	447.0942	447.0933	2.0	447.0949 (55) [M−H]^−^
								285.0391 (32) [M−H−gal]^−^
F7	9.128	kaempferol-3-*O*-glucuronide [35,41]	C_21_H_18_O_12_	[M−H]^−^	461.0733	461.0725	1.6	461.0741 (5) [M−H]^−^
								285.0397 (100) [M−H−glcA]^−^
F8	9.198	kaempferol-3-*O*-glucoside [35,41]	C_21_H_20_O_11_	[M−H]^−^	447.0940	447.0933	1.6	447.0961 (29) [M−H]^−^
								285.0404 (55) [M−H−glc]^−^
F9	9.332	quercetin-3-*O*-rhamnoside [35,41]	C_21_H_20_O_11_	[M−H]^−^	447.0938	447.0933	1.2	447.0950 (18) [M−H]^−^
								301.0351 (60) [M−H−rha]^−^
F11	10.358	kaempferol-3-*O*-rhamnoside [35,41]	C_21_H_20_O_10_	[M−H]^−^	431.0987	431.0984	0.8	431.1008 (26) [M−H]^−^
								285.0405 (88) [M−H−rha]^−^
								257.0458 (25) [M−H−rha−CO]^−^
F12	12.108	quercetin [35]	C_15_H_10_O_7_	[M−H]^−^	301.0366	301.0354	4.1	301.0360 (49) [M−H]^−^
								273.0406 (16) [M−H−CO]^−^
								245.0448 (8) [M−H−2CO]^−^
F13	14.125	kaempferol [36]	C_15_H_10_O_6_	[M−H]^−^	285.0418	285.0405	4.7	285.0408 (100) [M−H]^−^

glc: glucosyl; gal: galactosyl; rha: rhamnosyl; ara: arabinosyl; qui: quinovosyl; glcA: glucuronic acid; CA: caffeic acid; FA: formic acid; AcOH: acetic acid.

**Table 3:** Identification of the chemical constituents in CPT by HPLC-ESI-Q-TOF-MS/MS.

No.	t_R_ (min)	Identification	Formula	Selected Ion	Measured (*m*/*z*)	Calculated (*m*/*z*)	Error (ppm)	MS^2^ (*m*/*z*)
T14	16.787	3β,19α,23-trihydroxy-1-oxo-olean -12-en-28-oic acid	C_30_H_46_O_6_	[M−H]^−^	501.3235	501.3222	2.6	501.3294 (100) [M−H]^−^
								455.3225 (73) [M−H−FA]^−^
T15	20.616	arjunolic acid	C_30_H_48_O_5_	[M−H]^−^	487.3451	487.3429	4.5	487.3482 (100) [M−H]^−^
T16	22.005	cyclocaric acid B	C_30_H_46_O_5_	[M−H]^−^	485.3291	485.3272	3.9	485.3341 (100) [M−H]^−^
T17	22.160	asiatic acid	C_30_H_48_O_5_	[M−H]^−^	487.3454	487.3429	5.1	487.3483 (100) [M−H]^−^
T18	24.134	pterocaryoside B [37]	C_35_H_58_O_9_	[M−H]^−^	621.4029	621.4008	3.4	621.4104 (100) [M−H]^−^
								489.3639 (26) [M−H−ara]^−^
T19	25.328	cyclocarioside I	C_41_H_70_O_12_	[M−HCOO]^−^	799.4879	799.4849	3.8	799.5019 (25) [M+HCOO]^−^
								753.4958 (100) [M−H]^−^
								621.4500 (7) [M−H−ara]^−^
T20	25.683	cyclocarioside K	C_41_H_70_O_12_	[M−HCOO]^−^	799.4866	799.4849	2.1	799.5024 (20) [M+HCOO]^−^
								753.4952 (100) [M−H]^−^
								607.4322 (5) [M−H−qui]^−^
T21	25.895	pterocaryoside A [37]	C_36_H_60_O_9_	[M−H]^−^	635.4182	635.4165	2.7	635.4263 (100) [M−H]^−^
								489.3628 (20) [M−H−qui]^−^
T22	27.669	cyclocariol C [38]	C_30_H_50_O_5_	[M−HCOO]^−^	535.3671	535.3640	5.7	489.3636 (100) [M−H]^−^
T23	28.486	(20S,24R)-20,24-epoxy-25-hydroxy-12β -(α-L-arabinopyranosyloxy)-3,4-*seco*- dammara-4(28)-en-3-oic acid	C_35_H_58_O_9_	[M−H]^−^	621.4034	621.4008	4.2	621.4118 (100) [M−H]^−^
								489.3669 (16) [M−H−ara]^−^
T24	28.800	cyclocarioside B	C_42_H_72_O_12_	[M−HCOO]^−^	813.5038	813.5006	3.9	813.5178 (35) [M+HCOO]^−^
								767.5112 (100) [M−H]^−^
T25	29.028	hederagenin	C_30_H_48_O_4_	[M−H]^−^	471.3500	471.3480	4.3	471.3535 (100) [M−H]^−^
								453.3406 (11) [M−H−H_2_O]^−^
								425.3450 (9) [M−H−H_2_O−CO]^−^
T26	29.224	cyclocarioside H	C_43_H_72_O_13_	[M−HCOO]^−^	841.4989	841.4955	4.0	841.5109 (63) [M+HCOO]^−^
								795.5039 (100) [M−H]^−^
								735.4818 (47) [M−H−AcOH]^−^
T27	29.252	cyclocarioside X [39]	C_37_H_62_O_9_	[M−H]^−^	649.4344	649.4321	3.5	649.4446 (100) [M−H]^−^
								517.4000 (11) [M−H−ara]^−^
T28	31.790	cypaliuruside E [40]	C_36_H_58_O_8_	[M−H]^−^	617.4076	617.4059	2.8	617.4163 (100) [M−H]^−^
								471.3536 (13) [M−H−qui]^−^
T29	33.510	ursolic acid	C_30_H_48_O_3_	[M−H]^−^	455.3546	455.3531	3.4	455.3571 (100) [M−H]^−^

glc: glucosyl; gal: galactosyl; rha: rhamnosyl; ara: arabinosyl; qui: quinovosyl; glcA: glucuronic acid; CA: caffeic acid; FA: formic acid; AcOH: acetic acid.

## Data Availability

Not applicable.

## Sample Availability

Samples of the fractions are available from the authors.
